# Integration of Quantum Chemistry, Statistical Mechanics, and Artificial Intelligence for Computational Spectroscopy: The UV–Vis Spectrum of TEMPO Radical in Different Solvents

**DOI:** 10.1021/acs.jctc.2c00654

**Published:** 2022-09-27

**Authors:** Emanuele Falbo, Marco Fusè, Federico Lazzari, Giordano Mancini, Vincenzo Barone

**Affiliations:** † Scuola Normale Superiore di Pisa, piazza dei Cavalieri 7, 56126 Pisa, Italy; ‡ Dipartimento di Medicina Molecolare e Traslazionale, Università di Brescia, Viale Europa 11, 25123 Brescia, Italy

## Abstract

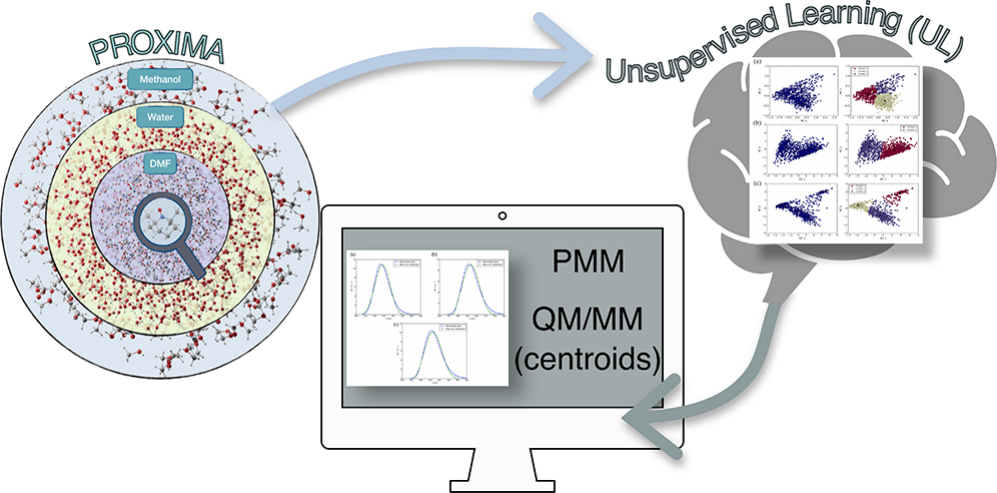

The ongoing integration of quantum chemistry, statistical mechanics, and artificial intelligence is paving the route toward more effective and accurate strategies for the investigation of the spectroscopic properties of medium-to-large size chromophores in condensed phases. In this context we are developing a novel workflow aimed at improving the generality, reliability, and ease of use of the available computational tools. In this paper we report our latest developments with specific reference to unsupervised atomistic simulations employing non periodic boundary conditions (NPBC) followed by clustering of the trajectories employing optimized feature spaces. Next accurate variational computations are performed for a representative point of each cluster, whereas intracluster fluctuations are taken into account by a cheap yet reliable perturbative approach. A number of methodological improvements have been introduced including, e.g., more realistic reaction field effects at the outer boundary of the simulation sphere, automatic definition of the feature space by continuous perception of solute–solvent interactions, full account of polarization and charge transfer in the first solvation shell, and inclusion of vibronic contributions. After its validation, this new approach has been applied to the challenging case of solvatochromic effects on the UV–vis spectra of a prototypical nitroxide radical (TEMPO) in different solvents. The reliability, effectiveness, and robustness of the new platform is demonstrated by the remarkable agreement with experiment of the results obtained through an unsupervised approach characterized by a strongly reduced computational cost as compared to that of conventional quantum mechanics and molecular mechanics models without any accuracy reduction.

## 1 Introduction

**Figure 1:**
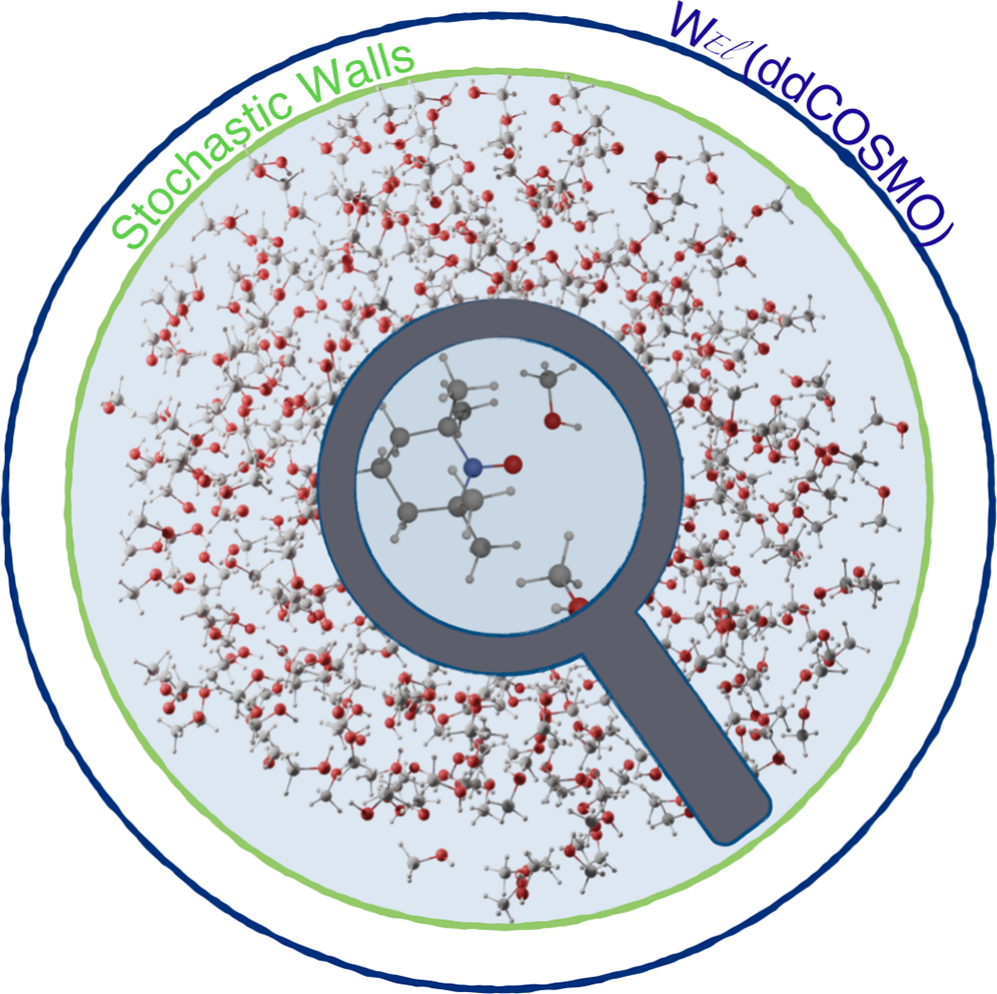
TEMPO radical in methanol solution. The spherical simulation box and the applied non periodic boundary conditions are also sketched.

Organic free radicals are usually highly unstable reactive species, with nitroxides being one of the few exceptions. This remarkable feature, together with the strong sensitivity of the structure and spectroscopic signatures of the NO moiety to both intra- and intermolecular environmental effects, has attracted considerable interest from experimental and theoretical points of view.^1,2^ In particular, nitroxides are widely used as spin labels and spin probes in both biological and material chemistry.^3−5^ The interest for this class of radicals has seen very recently a remarkable revival especially in connection with the analysis of dynamical and environmental effects by state-of-the-art computational approaches and with protein crystallography refinement.^6−8^ Moreover, while the EPR (electronic paramagnetic resonance) spectra of a huge number of molecular systems including the NO moiety have been recorded and interpreted by means of quantum chemical computations,^2,9−11^ the situation is different concerning electronic spectra. As a matter of fact, the one-photon absorption spectrum of nitroxides shows an intense π–π* band in the ultraviolet region, which is only marginally affected by solvent effects.^12^ On the other hand, the visible absorption band of nitroxides is related to the excitation of a lone-pair electron into an antibonding π* orbital, which, as usual for n−π* bands, is strongly sensitive to solvent polarity and solute–solvent specific interactions.^12,13^ Based on these premises, we have decided to investigate in detail the TEMPO radical (2,2,6,6-tetramethylpiperidin-1-yl)oxidanyl, see Figure 1), which represents a very suitable benchmark system in view of its scaffold rigidity, limited dimensions, and availability of experimental UV–vis spectra in several solvents.^13^

From a stereoelectronic point of view, different spectroscopic features of nitroxides are sensitive either to the NO bond length (g tensor) and/or to the piramidality of the N environment (hyperfine couplings),^14^ so that accurate structural characterization and proper description of the main vibrational modes (NO stretching and NO out-of-plane bending) are mandatory prerequisites for any accurate computational spectroscopic study.^15,16^ In recent years, integrated strategies combining accurate composite methods (for energies, properties and, when possible, geometries and harmonic frequencies) with last-generation hybrid and double-hybrid functionals (for anharmonic contributions together with structures and harmonic force fields when composite methods become too expensive) have been proposed and fully validated for a number of interesting situations.^9,16−18^ The accuracy of reference structures can be further enhanced by the recently introduced nano-LEGO approach, which employs well-chosen fragments (synthons) and linear regression analyses based on large databases of accurate structures.^19^

Next, solvent effects come into play, which require the generation of a sufficient number of representative structures by means of stochastic approaches, in the present context Molecular Dynamics (MD), possibly followed by suitable unsupervised learning procedures aimed to reduce the number of different structures and descriptive coordinates (features in Machine Learning jargon) needed to obtain well converged average results. In this connection, the fast and very effective polarizable continuum model (PCM)^20,21^ can be profitably employed in the absence of strong and specific solute–solvent interactions or to include bulk electrostatic effects in atomistic simulations employing non periodic boundary conditions (NPBC).^22−24^

Finally, reliable yet effective multiscale methods combining quantum mechanics and molecular mechanics (QM/MM) can be employed to compute averaged values of the sought properties.^25,26^ While in principle all the steps can be performed at the same time by the so-called extended *ab-initio* molecular dynamics,^27^ the time scales accessible to these approaches are at present too short to deliver converged results for medium-to-large size chromophores. Furthermore, the accuracy required in the computation of the spectroscopic parameters is usually much higher than that needed in the generation of the representative snapshots. In such circumstances, carefully parametrized molecular mechanics (MM) force fields^28,29^ or semiempirical methods^30^ can be profitably employed to sample the potential energy surface (PES) of interest, whereas the computation of accurate spectroscopic parameters requires refined QM descriptions of the solute (and, possibly, the nearest solvent molecules).^31^

Based on these premises, the initial atomistic simulation is performed by a new computational engine for classical MD employing a spherical simulation box^22,23^ and including a quasi-analytical implementation of reaction field effects at its outer boundary.^24^ In the present work we introduce a new unsupervised building of spherical simulation boxes from a library of solvent structures followed by placement of a solute molecule at the center of the box and deletion of all solvent molecules too close to any solute atom.^32^ Next, the selection of snapshots able to provide a well converged description of the trajectories is carried out with an in-house implementation of the Partition Around Medoids (PAM) algorithm.^33^ The feature space for the snapshot selection can be built automatically by a continuous perception of solute–solvent hydrogen bonds^32^ followed by a distance distribution analysis and a principal component analysis (PCA) and score evaluation of models with increasing dimension.

Then, QM/MM computations for a representative snapshot of each basin are performed, possibly including some solvent molecule in the QM region. Fluctuations within each basin are taken into account by an improved version of the cheap yet reliable Perturbed Matrix Method (PMM),^34,35^ which includes its latest developments,^36^ is directly linked to the Gaussian software,^37^ and employs as reference the representative cluster structure introduced above in place of the customary (and much less representative) chromophore in the gas-phase.^23,38^ Alternatively, an effective electric field from the solvent is obtained by averaging a limited number of representative snapshots selected by a new version of the Greedy Randomized Adaptive Search Procedure (GRASP).^24,28,39^ The implementation and comparison between those two approaches is an additional methodological novelty of the present work.

The integrated strategy sketched above has been employed to perform a comprehensive study of the TEMPO nitroxide radical in different solvents. To this end, after validating the selected methods rooted in the density functional theory (DFT) and its time-dependent extension (TD-DFT), the UV–vis spectra have been computed in different solvents. Since the whole procedure is cast in terms of a fully unsupervised workflow, together with the intrinsic interest of the specific system, the validation of the protocol paves the route toward the study of several other problems of current technological or biological interest.

## 2 Computational Methods

The first step is the definition of a suitable geometry of the solute by means of geometry optimizations at the revDSDPBEP86-D3BJ/jun-cc-pVTZ level (hereafter rDSD)^40−43^ including bulk solvent effects by the conductor version of the polarizable continuum model (CPCM).^44^ Since the CPCM results are very similar for all the three solvent considered in the present work (due to saturation effects above dielectric constants of about 20), the same geometry is used in all cases. More accurate structures are obtained by correcting the rDSD geometrical parameters with the linear regression approach (LRA).^45^ Within the latter, systematic errors affecting bond lengths and valence angles are corrected based on linear regressions, whose parameters were derived from a large database of accurate semiexperimental equilibrium geometries.^19^ Since the NO bond length is not included in the above database, we employ the difference between the rDSD and accurate value for the dimethylnitroxide radical.^24^ Atomic charges to be used in MD computations were obtained by the CM5 recipe^46^ from B3LYP/jul-cc-pVDZ^41,42,47^ Kohn–Sham orbitals and splitting the oxygen charges between the atomic center and two lone-pairs according to a previously described procedure.^38^

### 2.1 Generation of Simulation Spheres

The Proxima Molecular Perception library^32^ has been equipped with a new tool devoted to the automatic creation of simulation boxes for studies in condensed phases. Different solvents can be selected from a continuously updated library or an external file can be loaded, which contains the coordinates of the atoms in the repeating cell and the parameters of the latter. Then, the structure of the solute can be taken from a database of accurate semiexperimental equilibrium geometries or optimized at the rDSD level and then corrected by the accurate yet effective nano-LEGO approach.^19^ Once the periodic structure of the environment and the molecular structure of the solute have been loaded, it is possible to replicate the molecules of the environment so as to cover the space surrounding the solute up to a maximum distance (*r*_*max*_). In the case of simulations enforcing NPBC, it is necessary to select the environmental molecules occupying a sphere of radius *r*_*max*_ (chosen by the user) and centered at the solute center of mass. Since the solvent cell is defined by an origin vector and three generally nonorthogonal vectors oriented along the sides of the cell, it is possible to replicate molecules belonging to the environment along the three cell axes by finding the origin of the new cell that surrounds the sphere and then generating the proper number of replicas of the unit cell, along the axes, so as to cover the entire sphere. Solution of both problems (by the procedures described in the Supporting Information, SI) leads to the definition of the solvation box, which, however, has not yet the correct spherical symmetry and can include some solvent atoms too close to the solute. We then proceed to erase all the solvent molecules whose centroids lie above a given threshold from the solute or “too close“ (below a given threshold) to the solute. In the latter case we do not use the centroid of each molecule to test the condition, but rather individual atom positions and a default threshold corresponding to the sum of their van der Waals radii. For purposes of illustration, Figure 1 shows the simulation sphere generated for TEMPO in methanol solution.

### 2.2 NPBC Simulations

The MD simulations include the TEMPO solute and three different solvents, namely *N*,*N*-dimethylformamide (hereafter DMF), methanol, and water.

The framework of the whole computational strategy is the GLOB model^22^ in which a spherical cavity with rigid boundary contains the proper number of solvent molecules needed to enforce an average density equal to the experimental counterpart. Next, two soft potentials are added in order to describe the reaction field of the solvent outside the cavity (*U*_*RF*_) and to enforce a nearly constant density in the whole simulation sphere (*U*_*vdW*_) (see Figure 1). In the present context, we employed in all cases a spherical box with radius of 20 Å containing 1106 (water), 503 (methanol), or 231 (DMF) solvent molecules.

The topology of the TEMPO solute was adapted from the study of Stendardo et. al,^48^ discarding intramolecular interactions since we kept the molecular structure frozen in all the simulations. In this model two virtual sites are used to describe the lone-pairs of the nitroxide oxygen. The topology and force field parameters of the nonaqueous solvents were the same used in previous studies,^22^ whereas the details of the TIP3P-FB water models are given in ref (49).

All the MD simulations were run with a locally modified version of the Gaussian^37^ suite of programs employing in most cases a quaternion formalism to treat rigid-body fragments^50−52^ and using the rotational velocity Verlet (RVV1) integrator^24,53^ with a tight convergence criterion (ϵ = 10^–9^) for the calculation of quaternion derivatives. Only methanol was treated as a flexible molecule and simulated employing the standard velocity Verlet integrator and enforcing holonomic constraints by means of the RATTLE method.^54^ Reaction field effects at the outer boundary of the simulation sphere were taken into account by the ddCOSMO model.^24^ Mechanical restraints were imposed instead by “rough walls”, such that whenever a solvent molecule goes outside the simulation sphere, its center of mass (COM) velocity is aligned along a new direction sampled with uniform probability in a unit emisphere, with a new magnitude corresponding to the average velocity at the thermostat temperature, and the angular momentum is reset in the same way generating a new angular velocity.

All the starting structures were initially brought to a low-energy configuration with a conjugate gradient minimization. A time step of 4 fs and thermostat coupling constants of 0.4 ps (2 fs and 0.2 ps for methanol in view of its flexibility mentioned above) were employed. The different runs were started sampling initial COM velocities and angular momenta at 298.15 K and carried out for 12.5 ns, with the first 2.5 ns used only for equilibration and not entering the following analysis. The good equilibration achieved in this way is witnessed by the plots of kinetic, potential and total energies as functions of time reported in Figures S4, S5, and S6 of the SI for the MD simulations of TEMPO in water, methanol, and DMF, respectively.

Radial distribution functions (RDFs) were calculated using an in-house python program along with the MDTraj package.^55^ Coordination numbers of the NO moiety were then obtained as integrals of the pertinent RDFs up to their first peak.

The hydrogen bond analysis was performed by employing the F_*HB*_ function proposed in ref (56) and implemented in the Proxima library.^32^ Such a function depends on both the H_*s*_O distance (*R*) and the H_*s*_O*_s_*O angle (ϑ), where the subscript *s* refers to a generic solvent. The function assumes values in the interval [0,1] and is defined as follows:
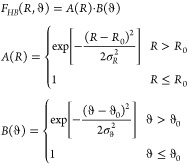
1The empirical parameters are *R*_0_ = 1.85 Å, σ_*R*_ = 0.20, ϑ_0_ = 0°, σ_ϑ_ = 10°, and values lower than 10^–4^ are considered as zeros.

### 2.3 Clustering and Trajectory Partitioning

The effective computation of electronic spectra from a classical MD simulation involves the selection of a reduced number of frames from the complete trajectory without any significant loss of information by means of unsupervised learning methods^57^ employing suitable feature spaces.^58^ In the present context the most significant features are the distances between the NO group (including the virtual sites associated with lone pairs (LP)) of TEMPO and first and second neighboring atoms of each solvent molecule. In details, the selected features are N–A_1_, N–A_2_, O–A_1_, O–A_2_, and LP1–A_1_, LP2–A_2_ distances, with A_1_ and A_2_ being solvent atoms, while LP1 and LP2 are the virtual sites of nitroxide oxygen. To avoid short time correlations in the clustering, a frame every 10 ps was sampled. The dimension of this data set was subsequently reduced by a PCA. A clustering analysis is then carried out to individuate natural groups in the feature space of input data, and return the corresponding representative frames, referred to as cluster centroids. The PAM algorithm^33^ has been used throughout to determine the best number of clusters. Its performance has been tested in several runs for values from 2 up to 20 in terms of the internal validation criteria of Silhouette Score (SI), Dunn Index (DI), and Calinski–Harabasz score (pSF).^59^ Furthermore, we accounted for a break-even point in the Within Sum of Squares error (WSS). SI, DI, and pSF should have a maximum corresponding to the parameter set (the value of *k* in this case) that yields the best clustering while WSS searches for a change in the slope. The best value of *k* was determined from the convergence of three criteria out of four. Their values can be found in the Supporting Information (see Figure S1)

Having obtained distinct simulation basins, the number of frames within each cluster can be further compressed by defining a minimum amount of equally relevant frames by a new implementation of the GRASP algorithm,^24,28,39^ which permits to obtain a number of frames proportional to the cluster sizes. Finally, both cluster centroids and GRASP frames are employed in the subsequent computation of electronic spectra.

### 2.4 Absorption Spectra

Absorption spectra in different solvents were computed by an integrated approach starting from PW6B95D3/SNSD^45,60^ (hereafter PW6) TD-DFT computations of TEMPO including the point charges of all solvent molecules for one representative frame in each basin. Then, for all the remaining frames of each basin, the perturbing effects exerted by solvent fluctuations are obtained either by the PMM or the collective frame approach.

In the PMM route, perturbed Hamiltonian matrices are built in the basis of the first 11 eigenstates obtained from the reference computation at the representative frame. To this end, a perturbation operator describing the difference of the electrostatic potential on the solute atoms between the reference and actual solvent configuration is added to the reference Hamiltonian, whose eigenvalues are then used to obtain perturbed excitation energies. Further details about the PMM approach are given in refs (23), (34)–^36^, and (61).

The second route permits, instead, to estimate the effect of solvent fluctuations by a single additional QM/MM computation of the absorption spectrum by means of a *collective frame* obtained by employing all the solvent molecules of *N* selected frames and assigning 1/*N* of the actual atomic charge to each solvent atom in the QM/MM calculation. For each cluster, *N* is proportional to the cluster size and the individual frames are selected by means of the GRASP algorithm.^24,28,39^

### 2.5 Refinement of the Results

The separation of the different trajectories in basins and the definition of suitable reference structures for each of them permits a further refinement of the results only for those reference structures assuming that the PMM or collective frame approaches mentioned above take care of the perturbations related to solvent fluctuations. A first refinement is to include a reduced number of solvent molecules in the part of the system treated at the QM level in order to introduce effects (e.g., Pauli repulsion, charge transfer, or polarization) lacking in the classical model used to describe the solvent. To this end, systematic computations were performed for the cluster centroids of the different MD simulations with increasing numbers of solvent molecules (*N*_*QM*_) included in the QM region in order to determine the smallest *N*_*QM*_ providing well-converged results.

From another point of view, classical MD simulations with rigid solutes do not include vibrational modulation (vibronic) effects in electronic spectra. On the grounds of previous experience, those effects are explicitly computed only for the reference structure of each basin by effective models based on the Franck–Condon principle. For a more detailed description of the approach, see ref (62).

Let us conclude this section by pointing out that the development of effective clustering and perturbative models permits to obtain fully converged results with a few (at most three in the present case) reference QM/MM computations followed by the same number of lower level collective frame QM/MM computations or by very cheap PMM steps in place of the hundreds of high-level computations required by traditional approaches.

## 3 Results and Discussion

### 3.1 MD Simulations

**Figure 2:**
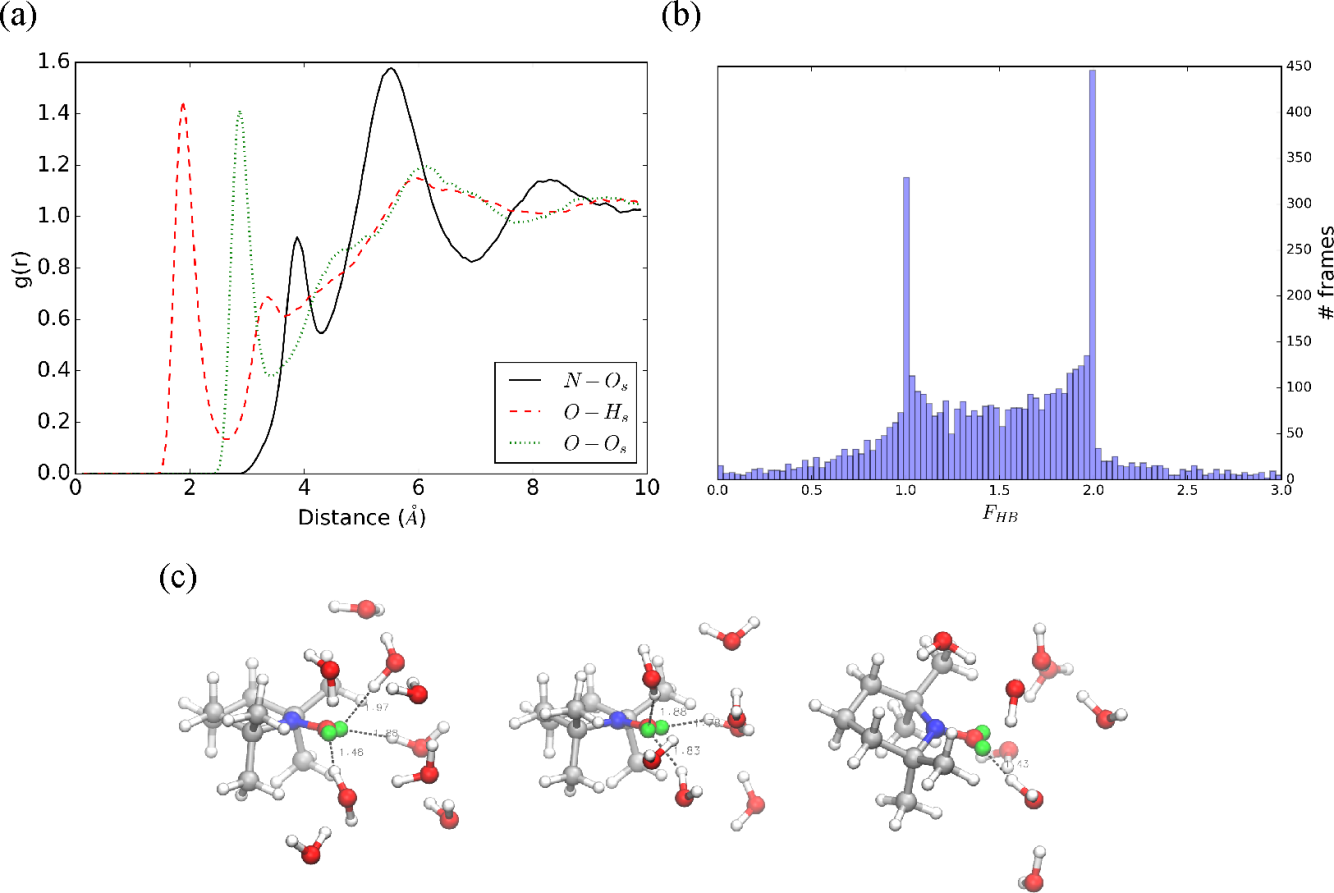
(a) TEMPO–H_2_O radial distribution functions for N–O_*s*_, O–H_*s*_, and O–O_*s*_ atom pairs. (b) histogram of the F_*HB*_ function. (c) Cluster centroids of TEMPO–H_2_O simulation.

The trajectory issued from the TEMPO–water simulation shows strong directional solute–solvent interactions, which are well represented by the positions of the first peaks of the RDFs, located at 3.88, 2.88, and 1.93 Å for N–O_*s*_, O–H_*s*_, and O–O_*s*_, with the subscript *s* used again for atoms belonging to solvent molecules. Next, well-defined depletion zones separate the first and second coordination shell for the O–H*_s_* and the O–O*_s_* pairs (see Figure 2a). This suggests that the peaks correspond to one and two solute–solvent hydrogen bonds, respectively with a continuous transition between the two most likely situations. As a matter of fact, the computed coordination number of the NO moiety is 1.9.

**Figure 3:**
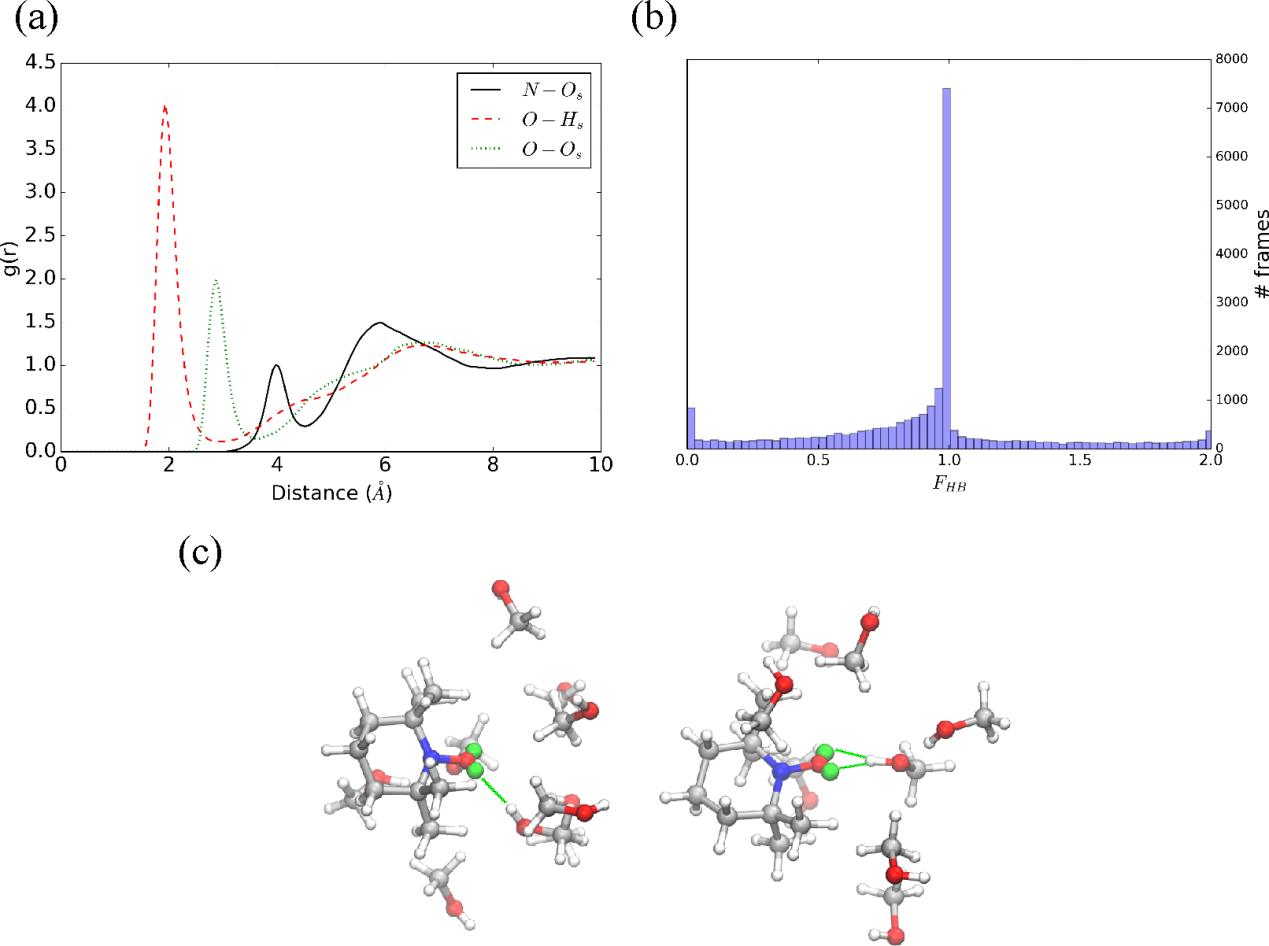
(a) TEMPO–CH_3_OH radial distribution functions for N–O_*s*_, O–H_*s*_, and O–O_*s*_ pairs. (b) Histogram of the F_*HB*_ function. (c) Cluster centroids of TEMPO–CH_3_OH simulation.

Also, the RDFs calculated from the simulation of TEMPO in methanol show sharp well-defined peaks and depletion zones for the atoms involved in the solute–solvent hydrogen bonds, with this pointing out that only one stable hydrogen bond is established during most of the simulation. The first maximum and minimum are located at 1.93 and 2.88 Å, respectively (see Figure 3a) and the computed coordination number is 1.0. Further information on the strength and dynamics of the hydrogen bond interactions can be obtained by using the *F*_*HB*_ function,^56^ whose histogram confirms that up to two stable hydrogen bonds are formed between TEMPO and water (see Figure 2b), whereas at most one stable hydrogen bond is formed between TEMPO and methanol (⟨*F*_*HB*_⟩ = 0.92) (see Figure 3b).

**Figure 4:**
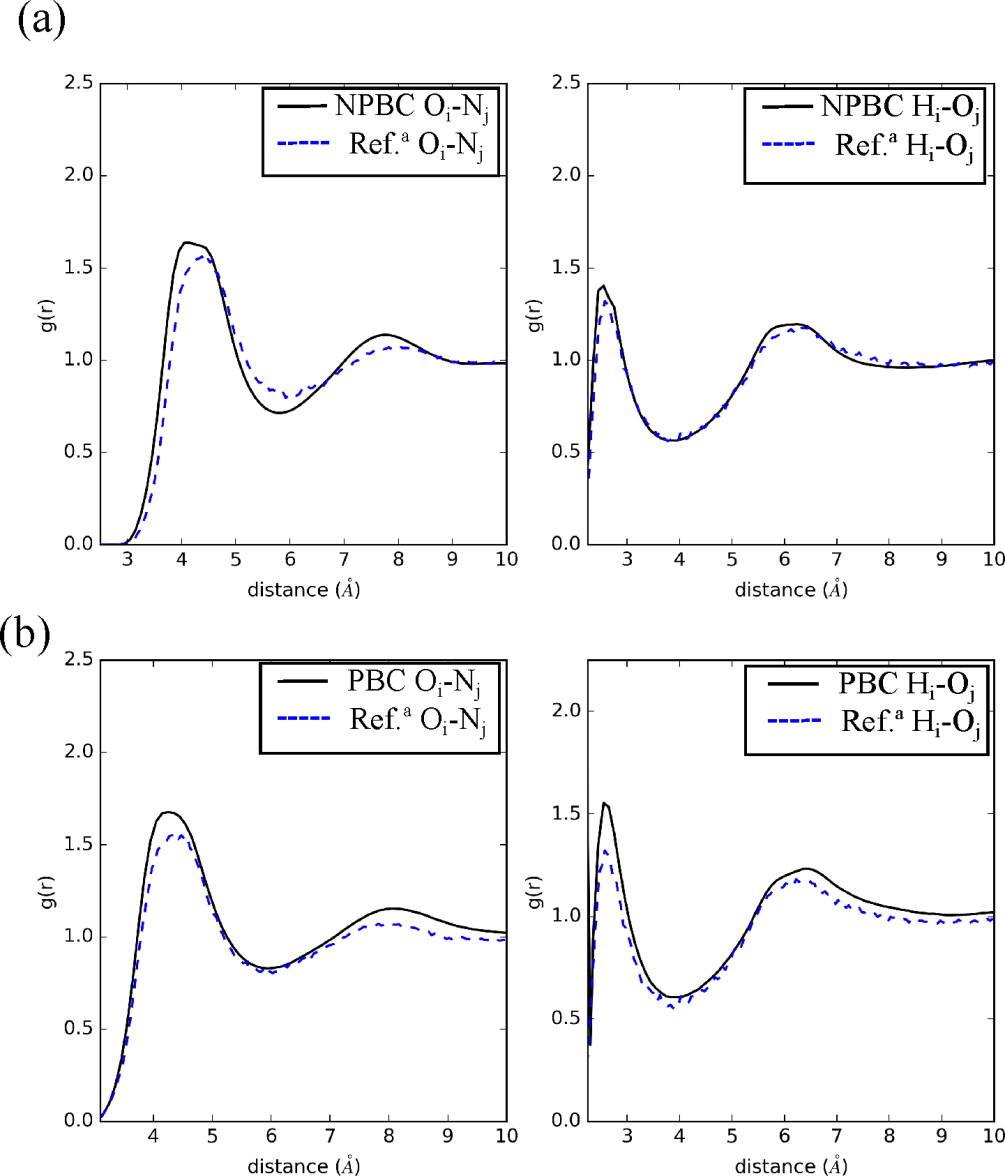
MD simulations of pure DMF. (a) Computed RDFs for N_*i*_–O_*j*_ and H_*i*_–O_*j*_ distances using an AA model with NPBC, i.e., methyl hydrogens explicitly taken into account; (b) RDFs obtained from an UA description with PBC. ^a^The results are directly compared with those of ref (63).

In the case of DMF, previous simulations were performed with a united atom (UA) model and employing periodic boundary conditions.^63^ As a consequence, we started from a pure liquid simulation employing an all atom (AA) model and our NPBC engine. In order to follow the evolution of the DMF structure in solution, we computed the O_*i*_–N_*j*_ and H_*i*_–O_*j*_ RDFs, with O_*i*_, N_*i*_, and H_*i*_ being the oxygen, nitrogen, and hydrogen atoms belonging to the HCNO moiety of the *i*th DMF molecule (see Figure 4). The O_*i*_–N_*j*_ RDF of pure DMF simulations shows a broad peak at 4.5 Å, which results from a weak interaction due to the interposition of a methyl group between nitrogen and oxygen atoms (Figure 4a). In contrast, a sharp peak at 2.3 Å is observed for the H_*i*_–O_*j*_ interaction (likely due to the formation of a relatively stable hydrogen bond^63^) followed by a broad shoulder at about 6.5 Å.

Inspection of Figure 4b shows that the RDFs obtained employing the UA representation in a NPBC simulation agree well with the corresponding PBC results.^63^ The direct comparison of AA and UA models shows that all the RDF profiles are very close, with the intensity of the AA peaks being only slightly higher than that of the UA counterparts. This difference can be ascribed to a more ordered liquid structure of DMF when the methyl hydrogens are explicitly taken into account.

**Figure 5:**
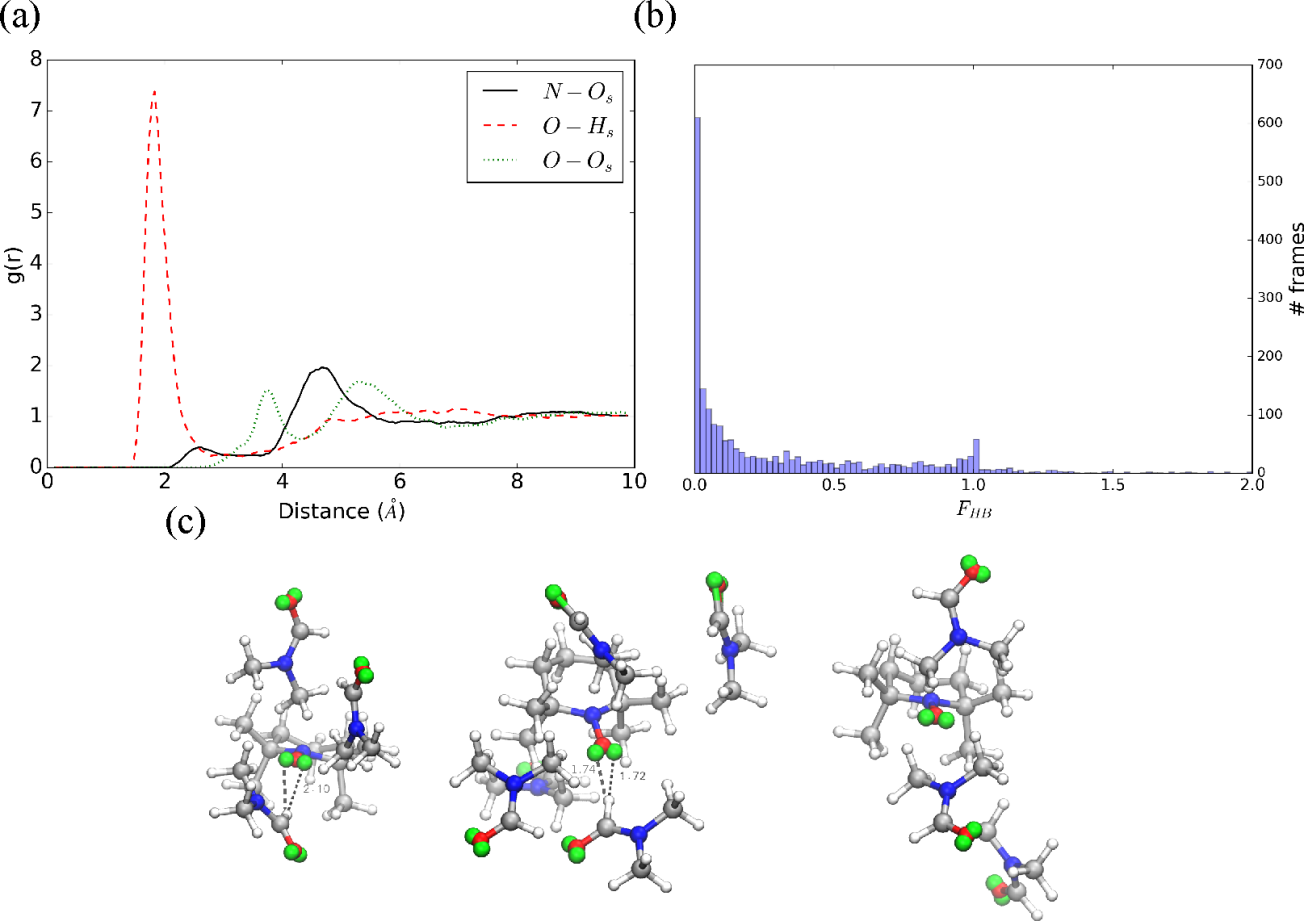
(a) TEMPO–DMF radial distribution functions for N–O_*s*_, O–H_*s*_, and O–O_*s*_ pairs. (b) histogram of the *F*_*HB*_ function. (c) Cluster centroids of TEMPO–DMF simulation.

Coming to the simulation of TEMPO in DMF, Figure 5a shows a sharp peak at 1.7 Å for the O–N_*s*_ RDF indicating the presence of significant solute–solvent interactions. However, the *F*_*HB*_ histogram shows that at most a single weak hydrogen bond is formed and in a reduced number of frames, as further confirmed by the low value of ⟨*F*_*HB*_⟩ (0.29). The N–O_*s*_ RDF shows a relatively intense peak at 4.7 Å, while two less defined peaks are observed for the O–O_*s*_ RDF. Their values suggest that steric effects due to the methyl groups of both solute and solvent lead the DMF molecules to approach the opposite plane of the methyl groups of TEMPO (Figure 5c).

In conclusion, the different MD simulations point out significant differences among the cybotactic regions (first solvation shells) of the TEMPO radical in different polar solvents, with the strength and average number of solute–solvent hydrogen bonds increasing sharply when going from DMF to CH_3_OH and to H_2_O.

### 3.2 Cluster Analysis

**Figure 6:**
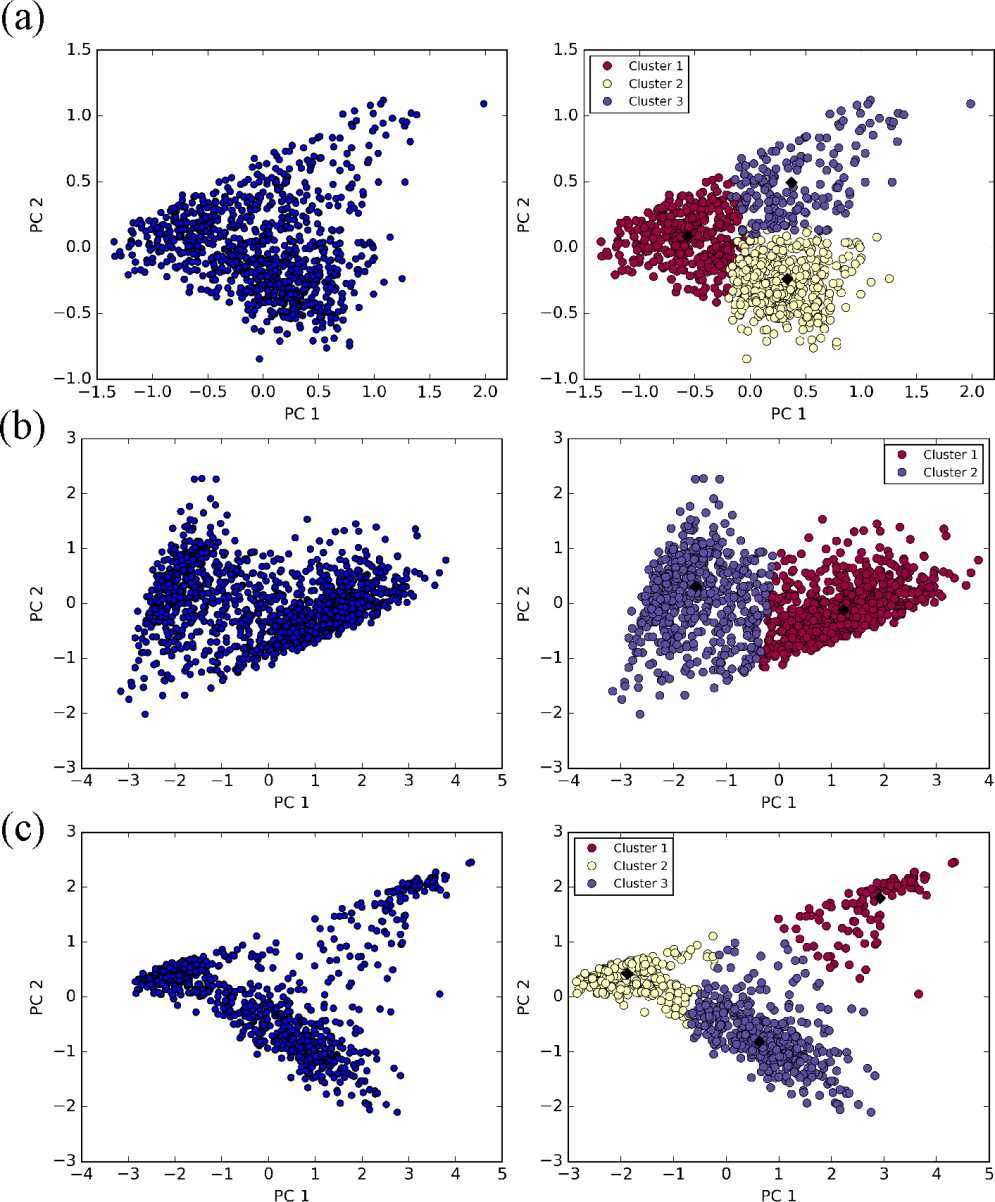
PCA plot of the two first components (left panels), and their corresponding clusters as obtained from the PAM algorithm (right panels) for (a) TEMPO in H_2_O (b) TEMPO in CH_3_OH, and (c) TEMPO in DMF. The centroids of each cluster are labeled by black diamonds.

A PCA of the feature space defined in section 2.3 for the TEMPO–water simulation shows that five components are necessary to account for 90% of the total variance and that three clusters can be defined containing 425, 343, and 173 frames, respectively (see Figure 6a). The first two clusters involve water molecules close to both sides of the nitroxide moiety, at distances compatible with the formation of hydrogen bonds and with some frames showing incipient exchange of solvent molecules. The last (and smallest) cluster shows a different structure with just one water molecule near a virtual site (see Figure 2c). The GRASP analysis produces 21, 17, and 8 representative frames for the three clusters.

For TEMPO in methanol, the PCA of the N–O, LP_1_–H, LP_2_–H, and O–O distances show that 93.1% of the total variance is accounted for when using the first three components, which lead to the definition of two clusters containing 702 and 548 points, respectively (see Figure 6b). The GRASP algorithm returns 35 and 27 representative frames for the first and second cluster, respectively, with a ratio (27/35 ≈ 0.77) reflecting the number of frames in each cluster (548/702 ≈ 0.78). Inspection of Figure 3 shows that the most populated cluster is characterized by stable hydrogen bonds oriented along one of the virtual site-oxygen bonds, whereas in the centroid of the less populated cluster the nearest methanol molecule lies between the two solute virtual sites suggesting that the included frames account for solvent exchange events.

In the case of TEMPO in DMF, the PCA reduction of a feature space analogous to that employed for TEMPO in methanol reaches 92% of the total variance with the first two principal components. Application of the PAM algorithm then produces three clusters containing 247, 205, and 70 frames, respectively. In agreement with the RDF results, the nitroxide oxygen is involved in the formation of weak hydrogen bonds in only one cluster, whereas solute–solvent hydrogen bonds are lacking in the other two clusters (Figure 5c).

## 4 Absorption Spectra

Let us now focus the attention on the computation of solvatochromic shifts in the absorption spectrum of TEMPO.

### 4.1 PCM Calculations

Table 1 compares the absorption maxima in the UV–vis spectra of the TEMPO radical in different solvents computed at the PCM/PW6 level with the available experimental counterparts. From a qualitative point of view, solvatochromic shifts are negligible for the UV band (with computed maximum near 237 nm), whereas this is not the case for the visible band (with the experimental maximum shifting from 475 up to 424 nm). As a consequence, in the following we will focus our attention only on the latter spectral region. From a quantitative point of view, the PCM/PW6 computational model performs a remarkable job for nonprotic solvents (hexane and DMF) providing results within 1.5 nm from the experimental counterparts. The situation is different for protic solvents (methanol and water), where the PCM approach (like any other continuum model) is not able to capture the specific effects leading to significantly different solvatochromic shifts for solvents (here DMF and methanol) with comparable dielectric constants. In order to check the sensitivity of the results on the specific density functional selected for the TD-DFT computations, we repeated the same computations at the B3LYP/SNSD level. Although some quantitative differences are observed (see Table S1 in the SI), all the general trends remain unchanged and, in particular, all polar solvents produce a nearly constant shift of the visible absorption maximum of about 10 nm with respect to hexane, whereas a negligible solvatochromism is again found for the UV absorption maximum. Furthermore, in all cases the absolute intensity (much larger for the UV than the visible band) consistently decreases with the solvent polarity, in agreement with experimental results.^13^

**Table 1:** Position (λ_*max*_ in nm) and Intensity (ε_*max*_ in M^–1^ cm^–1^) of the UV and Visible Absorption Maxima for the TEMPO Radical in Different Solvents Computed at the PW6B95/SNSD Level including Bulk Solvent Effects at the PCM Level and Obtained Experimentally; Values in Parentheses Are the Differences from the Values Obtained for Hexane

		Vis			UV	
		Calc.		Exp.	Calc.	
Solvent	Diel. Const.	λ_*max*_	ε_*max*_	λ_*max*_^a^	λ_*max*_	ε_*max*_
Gas phase	1.0	478.6	74.5		236.9	3097.4
Hexane	2.0	474.1	64.3	475.5	237.6	2437.8
CH_3_OH	32.6	464.1 (−10.0)	48.5	445.2 (−30.3)	237.7	1745.8
DMF	37.5	464.0 (−10.1)	32.2	464.1 (−11.4)	238.0	1088.4
H_2_O	78.4	463.5 (−10.6)	16.2	424.4 (−51.1)	237.7	455.4

a Ref (13).

In the following sections we will show that the differences between protic and non protic solvents can be described by atomistic simulations, provided that all the factors playing a role in the tuning of absorption spectra are taken into the proper account.

### 4.2 Setup and Validation of Effective Simulation Protocols

**Figure 7:**
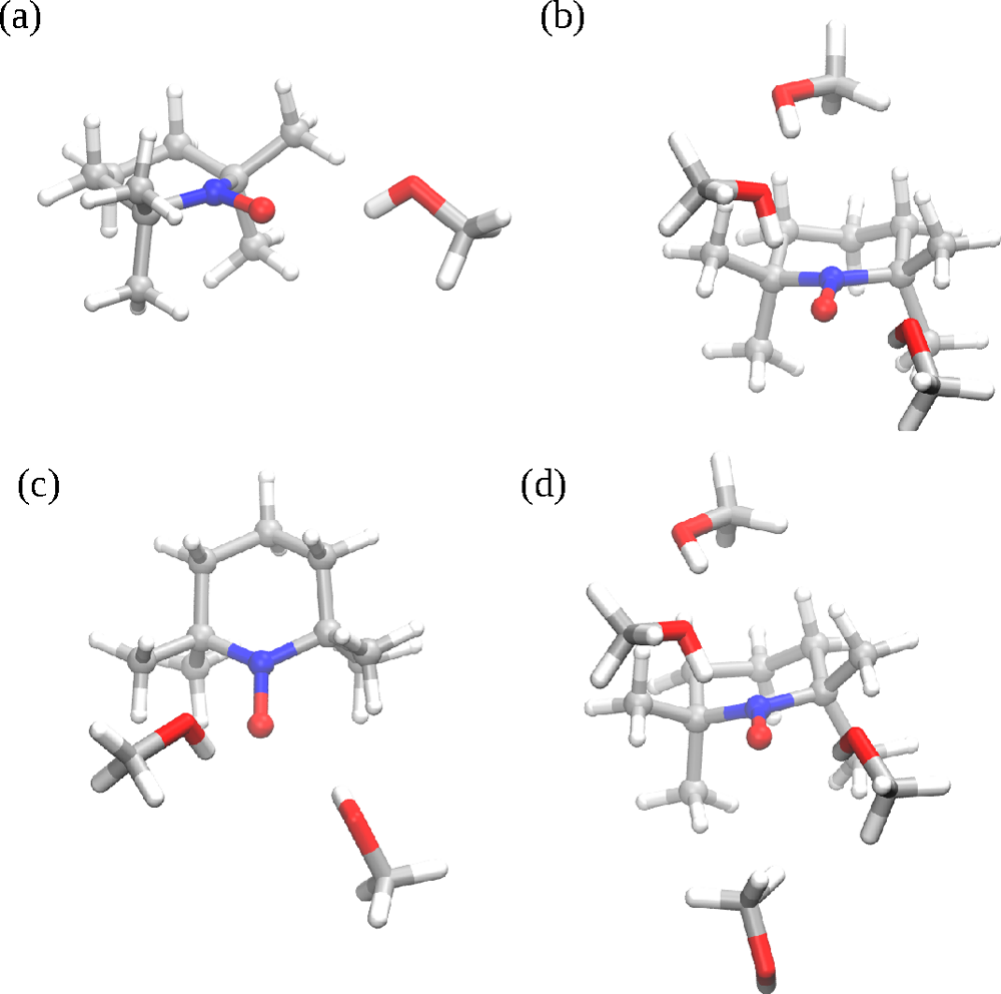
Representation of the centroid of cluster 1 issued from the TEMPO–methanol simulation with (a) 1, (b) 2, (c) 3, and (d) 4 CH_3_OH molecules included in the QM region of QM/MM calculations.

It has been previously reported that about 200 noncorrelated snapshots issued from MD simulations must be employed to obtain converged QM/MM results for spectra simulations.^63^ This will be our benchmark for setting up more effective approaches retaining the same accuracy of the reference computation. We will analyze in detail the methanol solvent, which is particularly challenging due to a strong difference of the solvatochromic shifts with respect to DMF, which has a comparable dielectric constant. Furthermore, either the bare solute or a small cluster including also some solvent molecules close to the NO moiety will be treated at the QM level. Starting from the bare solute, the absorption maximum averaged on 200 noncorrelated snapshots falls at 463.1 nm, to be compared with the value of 464.1 nm issued from the PCM computations. The close correspondence between the QM/MM and PCM results shows that the effect of explicit solute–solvent interactions is either negligible or not captured by description of solvent effects in terms of point charges. Deeper insight into this aspect has been gained by including an increasing number of solvent molecules in the QM system for the centroids of the two clusters of the TEMPO–methanol simulations described in a previous section. The results collected in Table 2 for the clusters sketched in Figure 7 show that converged results (i.e., within 1 nm from the best result) are obtained with two QM methanol molecules and that the absorption maximum is shifted by about 7 nm with respect to the corresponding computation in which all the methanol molecules are described by point charges. Analogous computations for water and DMF show the same trend (see Table 2), thus permitting to define a general computational approach in which improved results for reference frames are obtained including the pair of solvent molecules closest to the NO moiety in the QM region.

**Table 2:** Cluster Populations (Pop.) and Wavelengths of Visible Absorption Maxima (λ_*max*_ in nm) for the Different Clusters (*C*_*i*_) of TEMPO in Water, Methanol, and DMF with Increasing Numbers (*N*) of Solvent Molecules in the First Solvation Shell Treated at the QM Level and the Remaining Solvent Molecules Described by Point Charges

	Water			Methanol		DMF		
	C1	C2	C3	C1	C2	C1	C2	C3
Pop.	0.49	0.36	0.15	0.56	0.44	0.46	0.37	0.17
*N* = 0	441.2	438.6	425.4	455.3	465.9	462.0	466.5	440.0
*N* = 1	439.6	434.5	418.8	448.8	459.8	472.0	449.5	436.7
*N* = 2	436.9	435.8	418.8	448.3	458.9	472.6	449.8	435.9
*N* = 3	434.2	433.9	417.4	449.9	459.7	472.5	449.7	432.4
*N* = 4	434.6	432.9	418.9	449.4	459.8	–	–	–

On these grounds, we repeated the computations on the 200 noncorrelated frames of the TEMPO–methanol MD simulation discussed above always employing two QM methanol molecules. The final result (455.0 nm) differs by 8.1 nm from the counterpart lacking any QM solvent molecule. As a consequence, this contribution can be computed with sufficient accuracy just for the centroids of the trajectory clusters, with this allowing a reduction of 2 orders of magnitude (from 200 to 2) for the most expensive QM/MM TD-DFT computations.

Next, we compare two procedures for the effective inclusion of solvent fluctuations in the computed spectra of TEMPO in methanol. In the first one, the difference between the absorption spectrum computed for the centroid of each cluster and those of the other snapshots belonging to the same cluster are evaluated by the latest implementation of the inexpensive PMM approach described in section 2.4.^23,38^ The result issuing from this approach (460.2) is qualitatively correct, but not fully satisfactory from a quantitative point of view (3 nm lower than the reference value of 463.1 nm). In the second approach, the solvent configurations selected by the GRASP algorithm are treated as effective subtrajectories for each cluster, and their average effect is included in a single QM/MM computation of the absorption spectrum by means of a *collective frame* obtained by assigning 1/*N* of the actual atomic charge to each solvent atom in the QM/MM calculation, with *N* being the actual number of frames from each cluster. The absorption maximum for the average of the two collective frames (cointaining 36 and 28 structures, respectively) falls at 462.0 nm, i.e., within 1 nm from the counterpart obtained averaging 200 frames (463.1 nm) with a two-order of magnitude reduction of the computational effort. Noted is that the average of solvent charges for about 30 snapshots leads, even in the worst scenario, to the doubling of the computer time with respect to the use of solvent charges from a single snapshot. Adding to this result the solvent shift between 2 and 0 QM solvent molecules computed at the centroids of the clusters, we end up with a computed position for the absorption maximum (456 nm) again within 1 nm from the result of the reference computation employing all the 200 different snapshots. This gives full support to the claim that errors well within the most optimiztic error bars of this kind of computation can be obtained by our computational strategy, which is about two-orders of magnitude faster than the conventional one.

**Figure 8:**
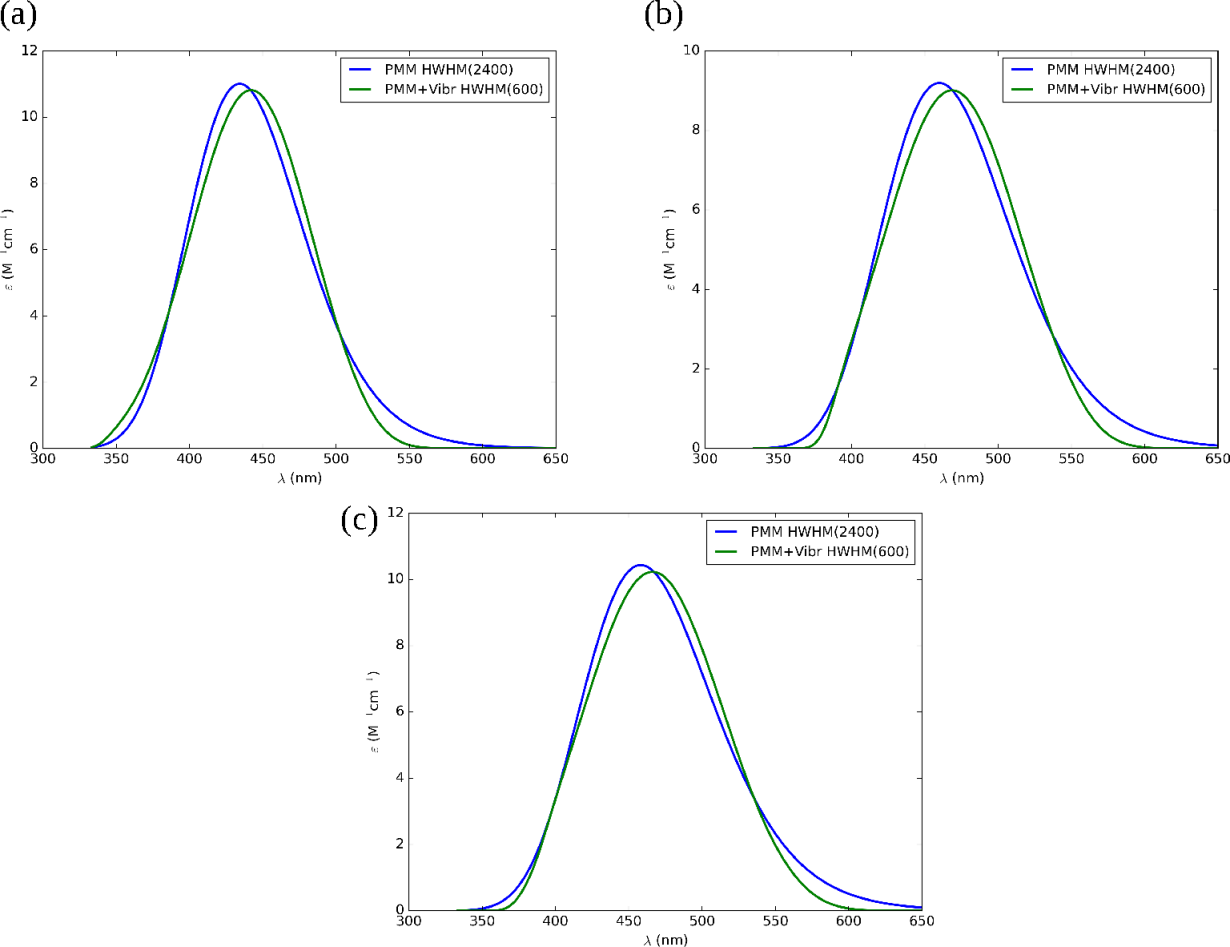
Electronic absorption spectra of TEMPO in (a) water, (b) methanol, and (c) DMF for PMM (blue) and vibrationally resolved PMM spectra (green). The spectra are averaged over the relative weights of their corresponding centroids. The different HWHMs are indicated in the legends.

The last aspect to be considered is related to the limitations of rigid solvent simulations and the neglect of vibrational averaging (vibronic) effects in the computed spectra. The renormalized vibrational contribution was added to the spectra of the different snapshots by means of a single vibronic calculation on the centroid of each cluster. A value of 600 cm^–1^ was used as (half-width at half-maximum) HWHM of the Gaussian distribution functions for the broadening. The absorption spectra computed including or not vibronic contributions are shown in Figure 8.

Vibronic contributions induce a displacement of the absorption maximum of about 7 nm for TEMPO in methanol, which should be included in the final result. However, analogous computations for the other solvents show that this contribution is nearly constant (e.g., 6.9 and 6.1 nm for water and DMF, respectively), so that it plays a negligible role in the evaluation of solvatochromic shifts.

### 4.3 Comparison of Spectra in Different Polar Solvents

**Figure 9:**
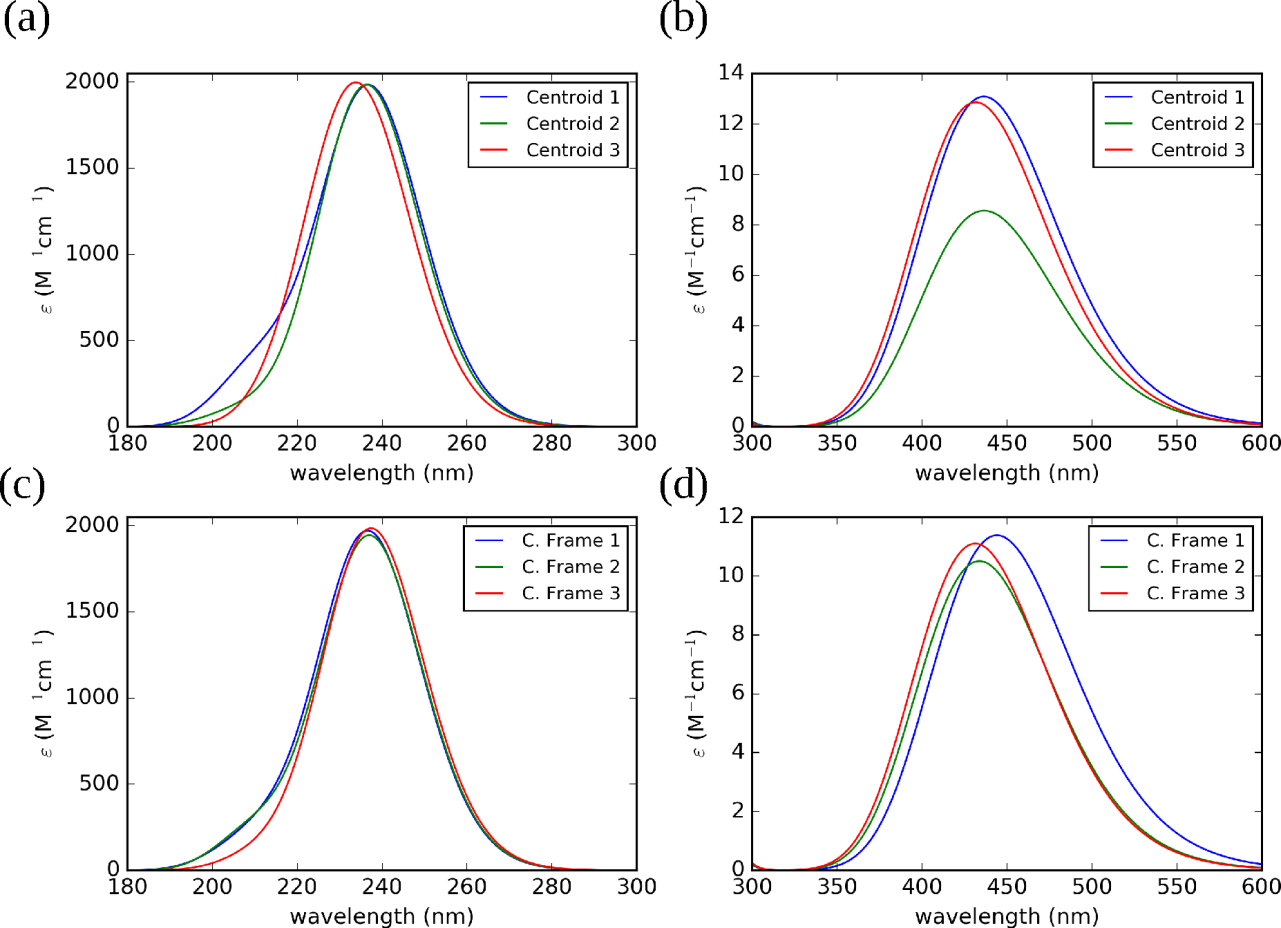
UV (a,c) and vis (b,d) absorption spectra for different clusters of TEMPO in water computed by the PMM using as references the cluster centroids (Centroids) or by the collective frame approach (C. Frame).

For purposes of illustration, Figure 9 shows the UV–vis electronic spectra of TEMPO in an aqueous solution computed with reference to the cluster centroids or collective frames. When the PMM is applied to the centroids the visible absorption maxima fall at 436, 436, and 431 nm for the cluster 1, 2, and 3, respectively, whereas the corresponding absorption maxima of the UV spectra fall at 236, 236, and 233 nm (see Figure 9b). Interestingly, the lower intensity obtained for the centroid of cluster 2 (8.5 M^–1^ cm^–1^) with respect to the other two centroids (13.0 and 12.8 M^–1^ cm^–1^ for cluster 1 and 3, respectively) parallels the less-ordered solvation shell around the NO group of TEMPO: as a matter of fact, the centroid of cluster 2 corresponds to the frame with only one solute–solvent hydrogen bond.

On the other hand, the intensities computed by employing the collective frame model are very close for all clusters: this behavior is not surprising when taking into account that the solvent effect is now averaged over several representative solvent configurations. The position of the absorption maximum is close (433 and 431 nm, respectively) for cluster 1 and 2, whereas a red-shift of about 9 nm is observed for the third cluster, with this suggesting that its subtrajectories sample regions more distant from the reference structure. In analogy with the centroid results, the UV spectra show comparable intensities of the absorption maximum at about 236 nm for all clusters (see Figure 9c).

**Figure 10:**
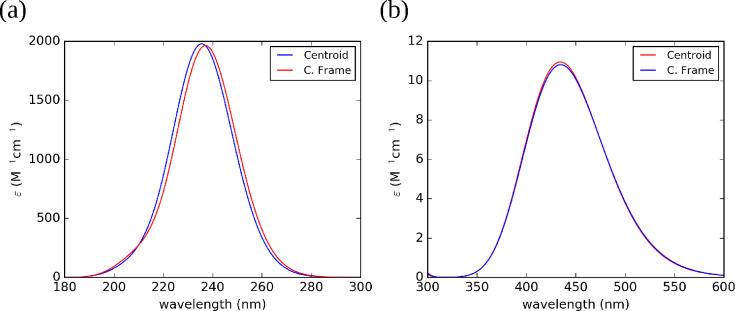
Fully averaged UV (a) and vis (b) absorption spectra of TEMPO in water computed by the PMM using as references the cluster centroids (Centroids) or by the collective frame approach (C. Frame).

The spectra obtained after averaging the results of the different clusters are shown in Figure 10. A comparison of Figures 9 and 10 confirms that the “collective frame” and “centroid” models lead to similar results, but the former approach is remarkably more effective and robust. The same trends are obtained for the absorption spectra in DMF and methanol (see Figures S8–S11 in the SI). Furthermore, all the absorption maxima in the UV region fall at about 236 nm (238.3, 236.3, 235.5 nm from the centroids and 236.6, 235.7, 237.0 nm from the collective frame for DMF, methanol and water, respectively) in fair agreement with the PCM results (237.7, 238.0, and 237.7 nm for DMF, methanol, and water, respectively) and with the value computed in the gas phase (236.9 nm). This confirms the expected insensitivity of the UV (π–π*) band to solvent effects.

The results discussed in the previous section suggest that the most effective procedure for computing absorption spectra in different solvents can be based on QM/MM computations for a reduced number of collective frames obtained by a Clustering/GRASP approach in which all the solvent molecules are represented by point charges. Next, the results are corrected by the difference of the results obtained including or not a reduced number of solvent molecules treated at the QM level for the centroids of the different clusters. When needed, vibronic contributions evaluated again for the different centroids can be added to the above results. The values obtained by this strategy for the different solvents are compared in Table 3 to the PCM (from Table 1) and experimental (from ref (13)) counterparts.

**Table 3:** Computed Solvatochromic Shifts (in nm) of the Visible Absorption Maximum of TEMPO Radical

	DMF solution			CH_3_OH solution		H_2_O solution		
dielectric constant (ϵ)	37.51			32.61		78.36		
Cluster number.	1	2	3	1	2	1	2	3
GRASP points	24	18	7	36	28	21	17	8
Weight of the cluster	0.49	0.36	0.15	0.56	0.44	0.46	0.37	0.17
1) *E*_*QM*/*MM*_ (coll. frame)	475.0	471.2	486.7	462.5	461.4	432.8	435.1	437.7
2) *E*_(*QM*+2)/(*MM*–2)_ (centroid)	472.6	449.8	435.9	448.3	458.9	436.9	433.9	418.8
3) *E*_*QM*/*MM*_ (centroid)	462.0	466.6	440.0	455.3	465.9	441.2	438.6	425.4
4) *E*_*PMM*_+Vibr.(centroid)	474.5	478.4	453.3	466.6	471.7	444.3	444.1	439.1
5) *E*_*PMM*_	465.5	469.3	445.1	458.1	462.8	436.4	436.4	431.6
Total^a^	494.9	463.5	490.7	464.0	463.2	436.4	438.0	438.6
Average^b^	483.0			463.6		437.7		
Δ^c^	0.0			–19.4		–45.3		
Δ(*PCM*)^c^^,^^d^	0.0			0.1		–0.5		
Δ(exp.)^c^^,^^e^	0.0			–18.9		–39.7		

a 1 + (2–3) + (4–5).

b Weighted average between the values of the different clusters.

c Shift w.r.t. DMF.

d Data from Table 1.

e Ref (13).

As already mentioned, the solvatochromic shifts computed at the PCM level for the three solvents (Δ(*PCM*))) are very close due to the poor description of specific solute–solvent interactions (in the present case H-bonds in methanol and, especially, water) and the saturation of reaction field effects for dielectric constants larger than about 20. On the other hand, the remarkable agreement between theory and experiment concerning the difference of solvatochromic shifts between protic and aprotic polar solvents shows that the proposed variational/perturbative strategy based on MD atomistic simulations is able to capture all the subtle tuning effects related to structural and dynamic properties of solute–solvent interactions.

## 5 Conclusions

In this paper we have extended a general molecular dynamics tool capable of enforcing non periodic boundary conditions, by adding an automatic generation of initial structures together with building of effective feature spaces for clustering of overall trajectories. Integration of a variational QM/MM approach for one representative structure belonging to each cluster and perturbative treatment of fluctuations within each cluster represents an optimal compromise between accuracy and computational burden. The proposed strategy has been tested for the challenging problem of the solvatochromic shifts in the UV–vis spectra of a prototypical nitroxide radical (TEMPO) in different solvents. The most remarkable result is that a very effective computational protocol for the proper inclusion of stereoelectronic and vibrational effects in the cybotactic region together with bulk solvent effects is able to settle the intriguing difference in the solvatochromic shifts of solvents with close dielectric constants. In more general terms, the good agreement of the results with the available experimental data confirms that we have at our disposal a robust and accurate tool for the study of electronic spectra of medium-to-large size systems in condensed phases. Further refinement of the computational strategy and extension to larger systems and other spectroscopic parameters under different conditions are under work in our laboratories.

## Supporting Information Available

Details about the building of spherical simulation boxes (including Figures S1–S3); temporal evolution of the energy during MD simulations (Figures S4–S6); details of the clustering of different MD trajectories (Figure S7); solvatochromic shifts computed at the TD-B3LYP/SNSD level (Table S1); computed spectra issued from different choices of reference configurations for TEMPO in methanol and DMF (Figures S8–S11) (PDF)
