# Supplementary material for: Integration of Quantum Chemistry, Statistical Mechanics, and Artificial Intelligence for Computational Spectroscopy: The UV–Vis Spectrum of TEMPO Radical in Different Solvents
Source: J Chem Theory Comput. 2022 Sep 27;18(10):6203–16. doi: 10.1021/acs.jctc.2c00654 (PMC9558374; doi:10.1021/acs.jctc.2c00654)
Supplement: Supplementary file 1 — ct2c00654_si_001.pdf [file ct2c00654_si_001.pdf]

Supporting Information: Integration of Quantum Chemistry, Statistical Mechanics and Artificial Intelligence for Computational Spectroscopy: the UV-vis spectrum of TEMPO radical in different solvents

Emanuele Falbo,† Marco Fusè,†,‡ Federico Lazzari,† Giordano Mancini,† and Vincenzo Barone*,†

†*Scuola Normale Superiore di Pisa, piazza dei Cavalieri 7, 56126 Pisa, Italy*

‡*Dipartimento di Medicina Molecolare e Traslazionale, Università di Brescia, Viale Europa 11, 25123 Brescia, Italy*

E-mail: vincenzo.barone@sns.it

Building of spherical simulation boxes

The problem of finding the origin of new cells surrounding the simulation sphere is solved by considering the three planes defined by the couples of vectors $(a, b), (b, c), (c, a)$ respectively (see Fig. S1 for the naming of the axes).

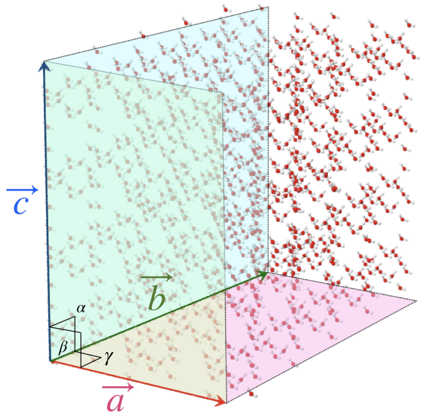

Figure S1: The $\vec{a}$, $\vec{b}$, $\vec{c}$ vectors define together the solvent cell. These are generally non orthogonal and their relative angles are $\alpha = \frac{\vec{b} \cdot \vec{c}}{||\vec{b}|| \cdot ||\vec{c}||}$, $\beta = \frac{\vec{a} \cdot \vec{c}}{||\vec{a}|| \cdot ||\vec{c}||}$, $\gamma = \frac{\vec{a} \cdot \vec{b}}{||\vec{a}|| \cdot ||\vec{b}||}$

Each of these planes defines two other tangent planes to the sphere by simple translation (See Fig. S2).

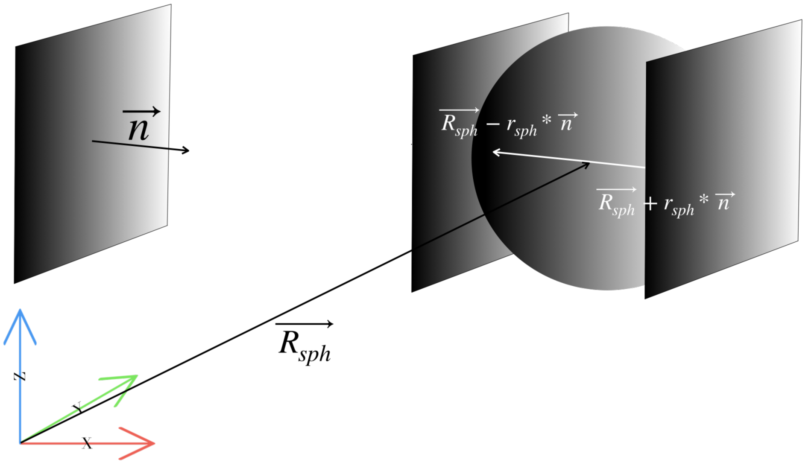

Figure S2: One plane defines two tangent planes to a sphere by simple translation. In the figure, $\vec{n}$ is the orthogonal vector to the plane that defines its orientation, $\vec{R_{sph}}$ is the center of the sphere, The tangent points are $\vec{R_{sph}} \pm r_{sph} \cdot \vec{n}$, where r_{sph} is the radius of the sphere.

To describe a plane, a vector perpendicular to the plane and an application point are needed. The perpendicular vectors are obtained by performing the cross products between the cell vectors (so $\vec{a} \times \vec{b}, \vec{b} \times \vec{c}, \vec{c} \times \vec{a}$). The application points are instead obtained by summing to the center of the sphere a vector with length equal to the radius of the sphere and parallel to the plane vector (see Fig. S2). Thus, since there are three cell planes and one sphere, there is a total of 3*2=6 tangent planes to the sphere. Then the origin of the new cell is obtained by intersection of the three planes having the following application points: $\vec{R_{sphere}} - \frac{\vec{a}\times\vec{b}}{||\vec{a}\times\vec{b}||} r_{sphere}$, $\vec{R_{sphere}} - \frac{\vec{b}\times\vec{c}}{||\vec{b}\times\vec{c}||} r_{sphere}$, $\vec{R_{sphere}} - \frac{\vec{c}\times\vec{a}}{||\vec{c}\times\vec{a}||} r_{sphere}$. This origin point is the $\vec{O}$ vector in Fig. S3.

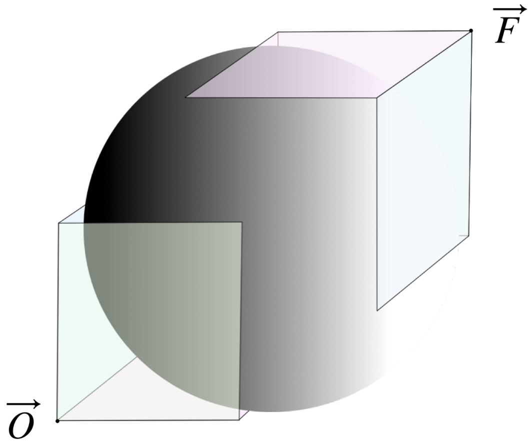

Figure S3: The $\vec{O}$ point is the origin of the new cell, the $\vec{F}$ point instead is the furthest away point from the origin of the cell. These points are obtained by simple intersection of the tangent planes to the sphere.

To solve the second problem instead, we first need to determine the most distant point of the new cell from the origin (the $\vec{F}$ point in Fig. S3). It has been already said that each plane defines two tangent planes to the sphere by simple translation. In order to find $\vec{F}$, we need to find the intersection between the planes with the following application points: $\vec{R_{sphere}} + \frac{\vec{a}\times\vec{b}}{||\vec{a}\times\vec{b}||} r_{sphere}$, $\vec{R_{sphere}} + \frac{\vec{b}\times\vec{c}}{||\vec{b}\times\vec{c}||} r_{sphere}$, $\vec{R_{sphere}} + \frac{\vec{c}\times\vec{a}}{||\vec{c}\times\vec{a}||} r_{sphere}$. The problem of finding the number of replicas of the cell along each axis so as to include the $\vec{F}$ point, in an orthogonal cell, would be simply solved by dividing the distance of the point from the origin by the

length of each side of the cell. The extension to non orthogonal reference axes requires the definition of a matrix having the cell axes as columns:

$$M = \begin{bmatrix} a_x & b_x & c_x \\ a_y & b_y & c_y \\ a_z & b_z & c_z \end{bmatrix} \tag{1}$$

Then we define the $\vec{r}$ vector as $\vec{F} - \vec{O}$. The number of replicas of the cell along each axis (l,m,n) is obtained by multiplying the inverse matrix of M by $\vec{r}$.

$$\begin{bmatrix} l \\ m \\ n \end{bmatrix} = M^{-1}\vec{r} \tag{2}$$

Once a solvent molecular system is loaded, all the atoms are translated so as to match the origin of the coordinates with the origin of the new cell ($\vec{O}$). Then, each solvent atom is duplicated $l \times m \times n$ times so as to cover all the space around the solute.

MD equilibration

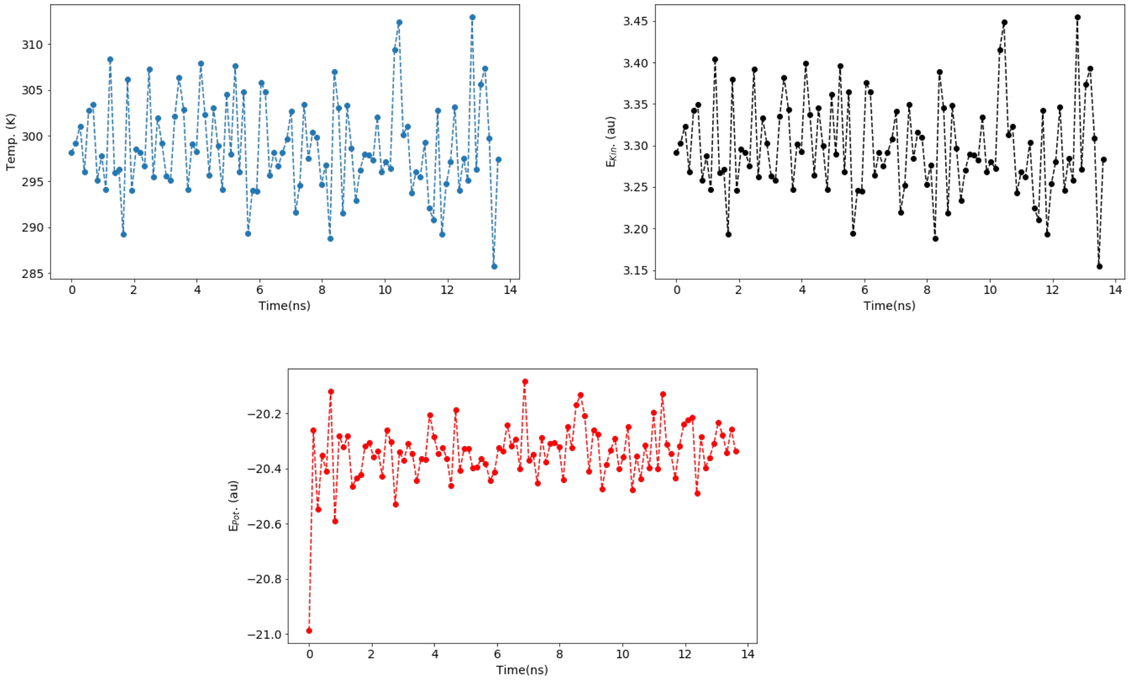

Figure S4: Kinetic, potential and total energy as a function of time for the MD simulation of TEMPO in water

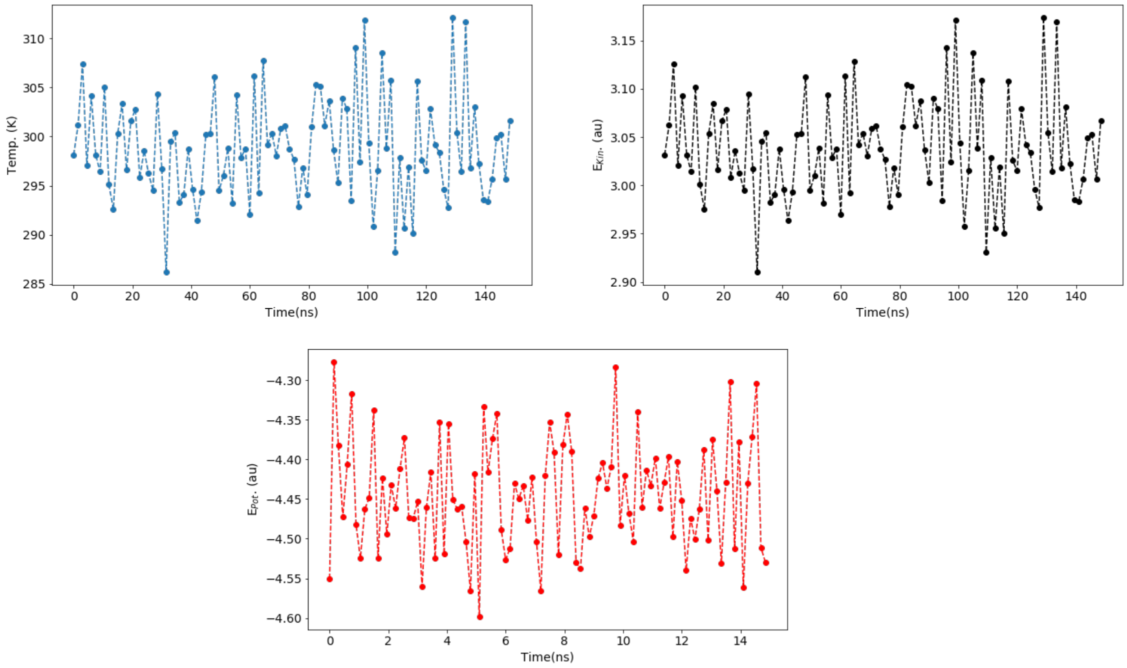

Figure S5: Kinetic, potential and total energy as a function of time for the MD simulation of TEMPO in methanol

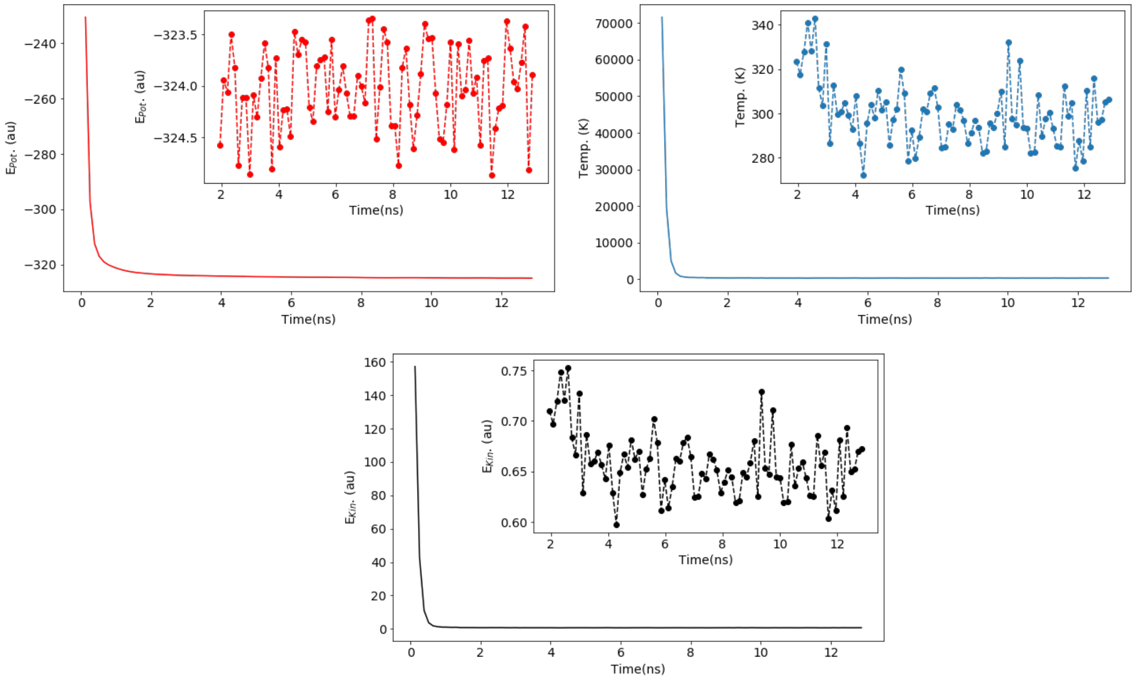

Figure S6: Kinetic, potential and total energy as a function of time for the MD simulation of TEMPO in DMF

Clustering

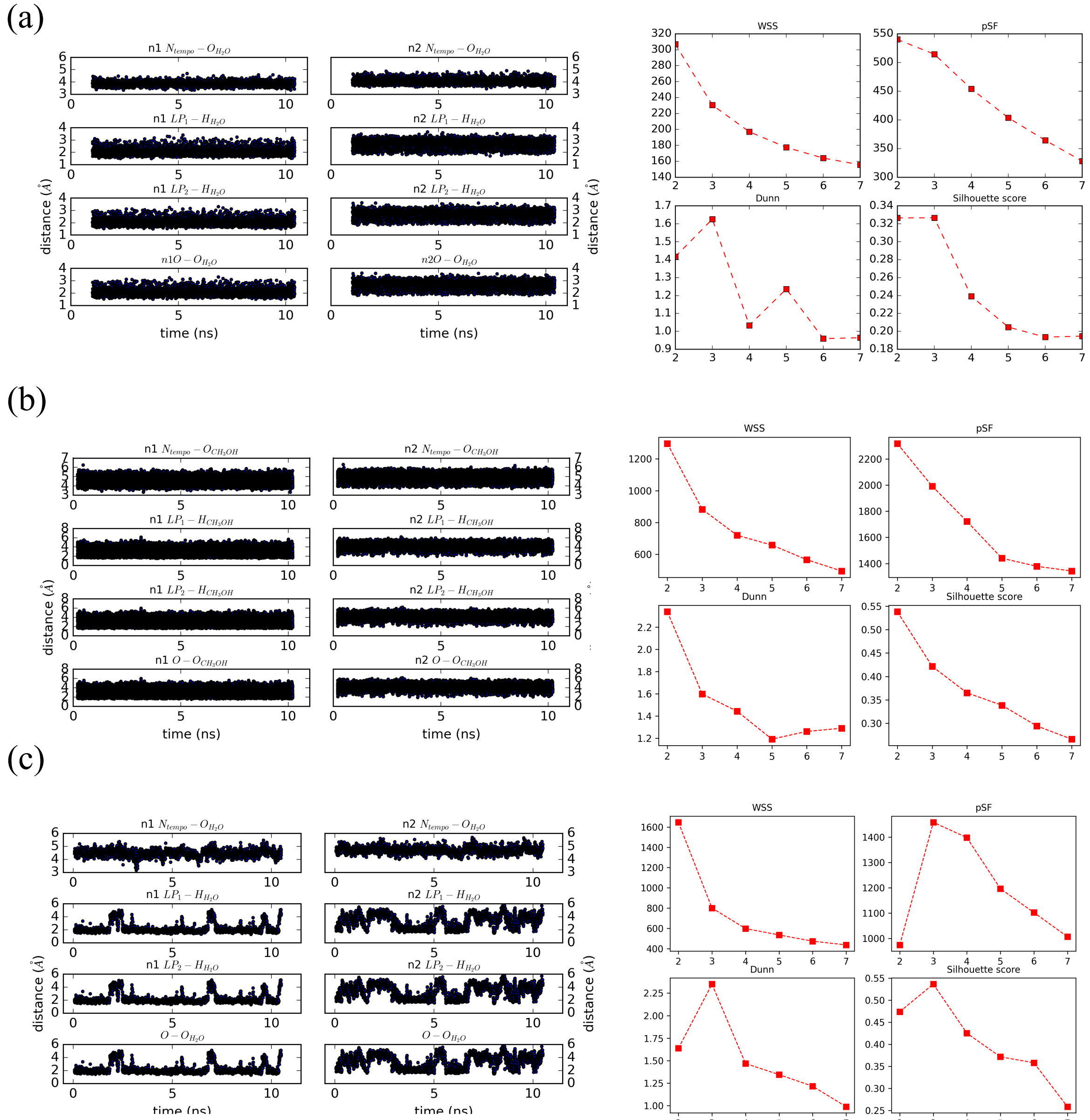

Figure S7: First and second nearest neighbour solute-solvent distances as a function of time (left panel), and within cluster sum of squares error (WSS), Calinski- Harabasz score (psf), Dunn Index (DI), and Silhouette coefficient (SI) computed for increasing k for **(a)** TEMPO-H_2O**(b)** TEMPO-CH_3OH, and **(c)** TEMPO-DMF simulations.

Adsorption spectra

Table S1: Wavelength of UV and vis absorbance maxima (λ_{max} in nm) for the TEMPO radical in different solvents computed at the B3LYP/SNSD level including bulk solvent effects at the PCM level and obtained experimentally. The values in parenthesis are the differences from the value computed in hexane. The molar extinction coefficients at maximum absorption (ε_{max}) are in M^{-1} cm^{-1}.

**Solvent**	**Diel. Const.**	**Vis**			**UV**	
		Calc.		Exp.	Calc.	
		λ_{max}	ε_{max}	λ_{max}	λ_{max}	ε_{max}
Gas phase	1.0	463.4	74.7		238.8	2257.1
Hexane	2.0	460.5	58.6	475.5	239.7	2042.4
CH_3OH	32.6	450.7 (-9.8)	42.6	445.2 (-30.3)	239.6	1665.7
DMF	37.5	450.7 (-9.8)	26.9	464.1 (-11.4)	240.5	1047.7
H_2O	78.4	450.2 (-10.3)	10.7	424.4 (-51.1)	240.2	447.9

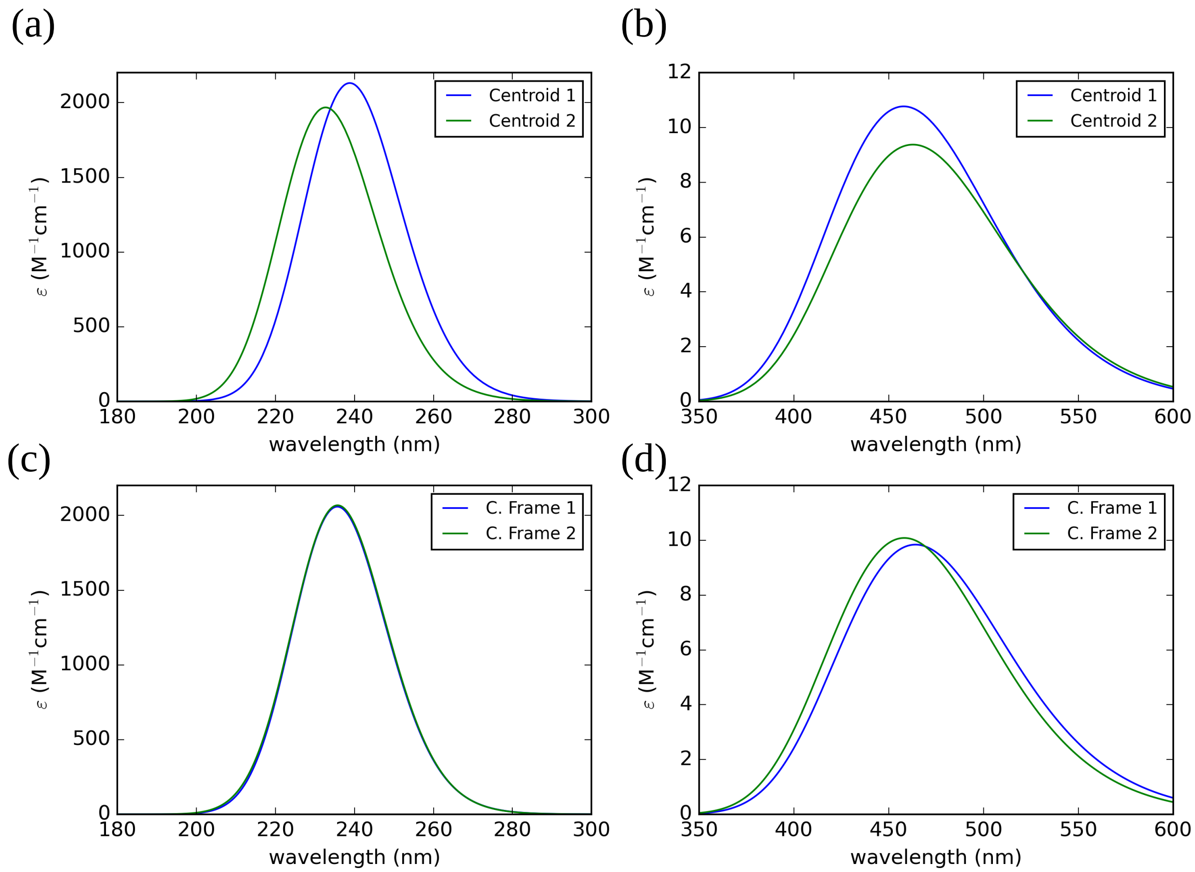

Figure S8: UV **(a,c)** and vis **(b,d)** absorption spectra for different clusters of TEMPO in methanol computed by the PMM using as references the cluster centroids (Centroids) or by the collective frame approach (C. Frame).

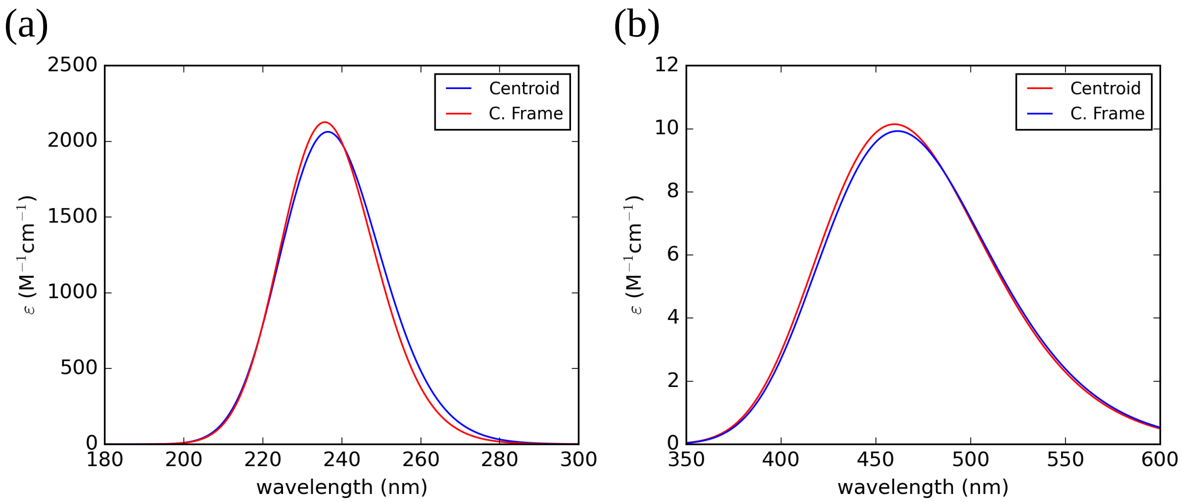

Figure S9: Fully averaged UV **(a)** and vis **(b)** absorption spectra of TEMPO in methanol computed by the PMM using as references the cluster centroids (Centroids) or by the collective frame approach (C. Frame).

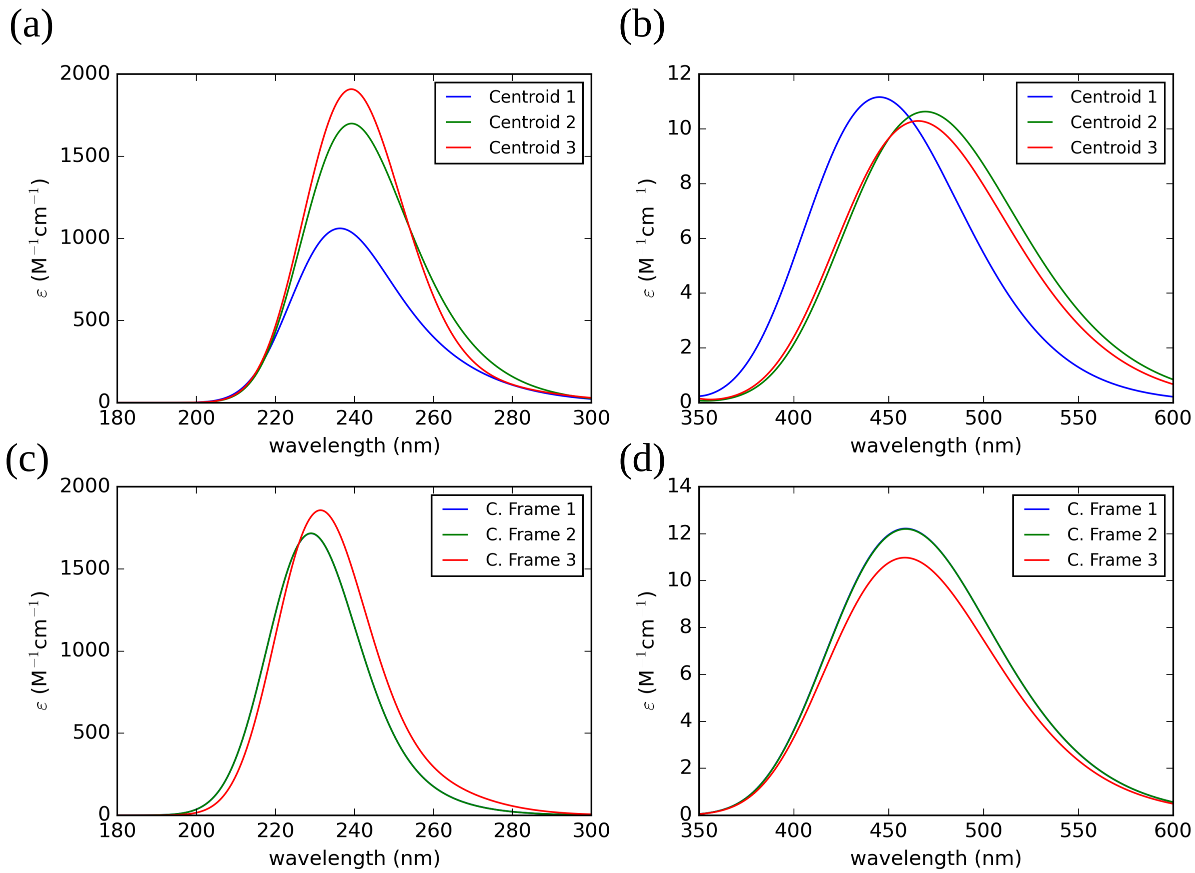

Figure S10: UV **(a,c)** and vis **(b,d)** absorption spectra for different clusters of TEMPO in DMF computed by the PMM using as references the cluster centroids (Centroids) or by the collective frame approach (C. Frame).

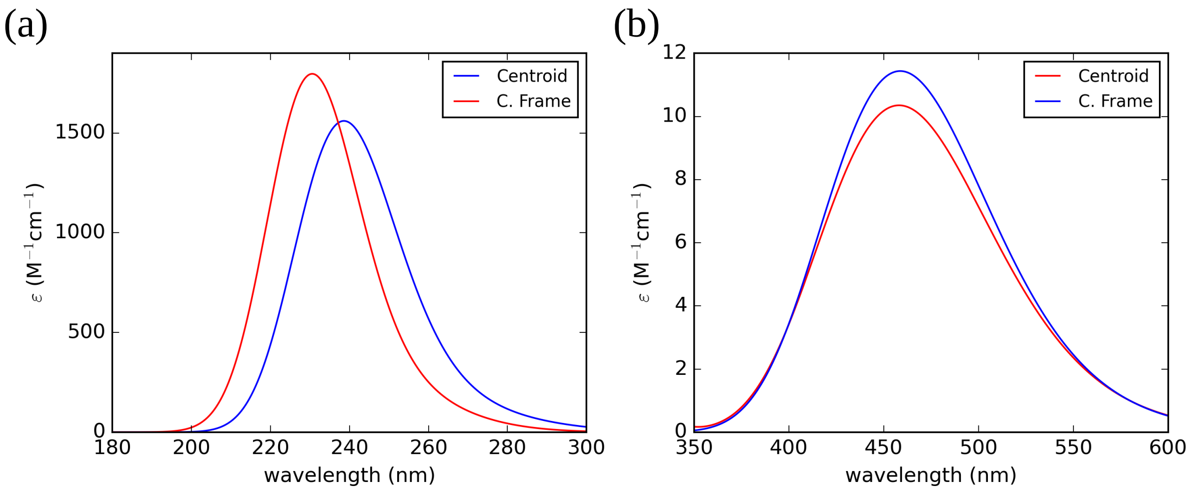

Figure S11: Fully averaged UV **(a)** and vis **(b)** absorption spectra of TEMPO in DMF computed by the the PMM using as references the cluster centroids (Centroids) or by the collective frame approach (C. Frame).
